# Adjuvant (chemo)radiotherapy for patients with head and neck cancer: can comorbidity risk scores predict outcome?

**DOI:** 10.1007/s00066-024-02282-y

**Published:** 2024-09-02

**Authors:** Sebastian N. Marschner, Cornelius Maihöfer, Richard Späth, Erik Haehl, Daniel Reitz, Nora Kienlechner, Lars Schüttrumpf, Philipp Baumeister, Ulrike Pflugradt, Julia Heß, Horst Zitzelsberger, Kristian Unger, Claus Belka, Franziska Walter

**Affiliations:** 1 Department of Radiation Oncology, University Hospital, LMU Munich, Munich, Germany; 2 Clinical Cooperation Group ‘Personalized Radiotherapy in Head and Neck Cancer’, Helmholtz Zentrum München, German Research Center for Environmental Health GmbH, Neuherberg, Germany; 3 German Cancer Consortium (DKTK), partner site Munich, a partnership between DKFZ and University Hospital Munich, Munich, Germany; 4 Department of Otorhinolaryngology-Head and Neck Surgery, Ludwig-Maximilians-University, Munich, Germany; 5 Research Unit Translational Metabolic Oncology, Institute for Diabetes and Cancer, Helmholtz Zentrum München Deutsches Forschungszentrum für Gesundheit und Umwelt (GmbH), Neuherberg, Germany; 6 Bavarian Cancer Research Center (BZKF), partner site LMU Munich, Munich, Germany

**Keywords:** Head and neck cancer, Comorbidities, Adult Comorbidity Evaluation-27 (ACE-27), ECOG-Performance status, American Society of Anaesthesiologists-Score (ASA-Score)

## Abstract

**Purpose:**

This study compares the objective American Society of Anesthesiologists (ASA) and Adult Comorbidity Evaluation-27 (ACE-27) scores with the subjective Eastern Cooperative Oncology Group performance status (ECOG PS) for patient outcome prediction.

**Methods:**

We retrospectively analyzed head and neck squamous cell carcinoma patients treated with adjuvant (chemo)radiotherapy at the LMU Munich from June 2008 to June 2015. The study focused on associations between patient outcomes; treatment failures; known risk factors (including human papillomavirus [HPV] status and tumor stage); and the comorbidity indices ECOG-PS, ASA score, and ACE-27. The Kaplan–Meier method and Cox proportional hazards model were used for survival analysis and identifying independent risk factors.

**Results:**

A total of 302 patients were analyzed, 175 received concurrent chemotherapy. Median follow-up was 61.8 months, and median age at diagnosis was 61 years. The 3‑ and 5‑year overall survival (OS) and disease-free survival (DFS) rates were 70.5%/60.2% and 64.7%/57.6%, respectively. Both ACE-27 and ASA showed significant correlations with OS in univariate and multivariate analyses, while ECOG-PS was significant only in univariate analysis. ASA and ACE-27 scores were also significantly correlated with local and locoregional recurrence, but only HPV status and tumor stage were significant in multivariate models.

**Conclusion:**

ACE-27 and ASA score effectively categorize patients’ risks in adjuvant radiotherapy for head and neck cancer, proving more predictive of overall survival than ECOG-PS. These results underscore the importance of objective comorbidity assessment and suggest further prospective studies.

**Supplementary Information:**

The online version of this article (10.1007/s00066-024-02282-y) contains supplementary material, which is available to authorized users.

## Background

Precise assessment of a patient’s performance status is crucial for adequate treatment decision making in patients with head and neck squamous cell carcinoma (HNSCC) undergoing surgery and adjuvant radio(chemo)therapy.

The Eastern Cooperative Oncology Group performance status (ECOG-PS) score was developed specifically for oncological patients in 1960 [1]. It rates patients in six groups ranging from ECOG-PS 0 (fully active) to ECOG-PS 5 (dead). In various analyses, ECOG-PS has shown its correlation with overall survival (OS) [2, 3] and cancer-specific survival (CSS) in HNSCC patients [4] but also in other oncological entities [5–7]. The simplicity of the ECOG-PS, while still being relevant in terms of outcome, has made it one of the most widely used tools in radiation oncology [8].

For specific use, more refined risk scores have subsequently been developed. Following initial surgery, the standard treatment for locally advanced HNSCC is adjuvant (chemo)radiation, with high local control rates of 90% after 3 years [9]. However, these patients often present with multiple significant comorbidities [10] such as coronary heart disease, diabetes, obesity, chronic obstructive pulmonary disease (COPD), or congestive heart failure, often due to tobacco and alcohol abuse. Depending on their severity, these comorbidities can also influence the long-term prognosis [11–14]. Unfortunately, the ECOG-PS primarily assesses the overall condition of a patient, which may not account for specific comorbidities. Additionally, the patient’s condition can be temporarily influenced by postsurgical side effects. This variability—where the general condition is altered, or new comorbidities appear shortly after surgery—can lead to inaccuracies in categorizing the patient’s overall condition when the ECOG-PS is applied postoperatively. This highlights potential limitations of the scoring system in such scenarios. While adjuvant (chemo)radiotherapy is highly efficient, it can cause severe side effects especially in patients with preexisting morbidities. Therefore, we hypothesize that the ECOG-PS assessed before adjuvant treatment is not the best available risk score for this treatment decision.

We identified two clinical risk scores that include typical comorbidities and clinical status before or independently of surgery and which could therefore potentially help to guide treatment decisions.

The ASA score was developed by the American Society of Anesthesiologists (ASA) to assess perioperative risks and the fitness of the patient the day before surgery with a rating of 1 (healthy) to 6 (dead). Besides more general criteria such as smoking, alcohol abuse, diabetes, and obesity, the focus here is on pulmonary and cardiac pre-existing conditions such as previous heart attacks, COPD, or aneurysms [15]. The ASA score has shown a high predictive value in peri- and postoperative treatment [14, 16, 17].

The Adult Comorbidity Evaluation-27 score (ACE-27) is based on the Kaplan–Feinstein index for patients with diabetes mellitus and has been specifically adapted and validated for tumor patients [14]. It requires a more sophisticated assessment that ranks patients according to the severity of 27 different comorbidities, including the most debilitating diseases of multiple organ systems, making it relatively independent of previous surgical procedures and short-term side effects. Besides classic cardiac, renal, gastrointestinal, and pulmonary diseases, it also includes psychiatric, immunological, or endocrine diseases [13, 18, 19]. The ACE-27 score hereby captures the broadest range of comorbidities of the three indices discussed but is also the most elaborate to determine.

We retrospectively analyzed 302 consecutive HNSCC patients who underwent adjuvant (chemo)radiotherapy in our department. The aim of the study was to find out whether the ECOG score alone is adequately prognostic for survival or local control or whether additional scores, such as the ASA score or ACE-27, should be used for guiding treatment decisions.

## Methods

Patients treated for newly diagnosed squamous cell carcinoma of the oral cavity, oropharynx, hypopharynx, and larynx between June 2008 and June 2015 in our institution were evaluated. We included all patients who underwent surgical resection and adjuvant (chemo)radiotherapy. All available patient data were reviewed in our clinical database, including the patient’s clinical history, comorbidities, initial tumor stage, human papillomavirus (HPV) status, and treatment parameters.

### (Chemo)radiotherapy

Patients with known risk factors such as positive lymph nodes (>pN1), large tumors (pT3/pT4), and close (< 5 mm) or incomplete resection received postoperative radiotherapy. Tumor stage was assessed using the UICC 7th edition classification.

The applied dose was usually 64–66 Gy to the operative bed, 56–60 Gy to the involved lymph node levels, and 50–54 Gy to the elective nodal levels applied via a 3D or intensity-modulated radiotherapy (IMRT) technique. Patients with extracapsular extension (ECE) of the involved lymph nodes (ECE+) and close or incomplete resection status additionally received concomitant chemotherapy, either cisplatin/5-fluorouracil (CDDP/5-FU; in accordance with the ARO 96–3 study), 5‑FU/mitomycin C (MMC; 10 mg/m^2^ d1, d29; 5‑FU 600 mg/m^2^ d1–5), cisplatin mono (40 mg/m^2^ weekly), or cetuximab mono (250 mg/m^2^ weekly with 400 mg/m^2^ loading dose).

### HPV

As a surrogate marker for HPV infection, immunohistochemical (IHC) p16^INK4a^ staining results from our local pathology institute and the framework of the clinical cooperation group were used [9, 20].

### Performance scores

Three different performance scores were determined for each patient. ASA score and ECOG-PS were assessed as part of the clinical treatment routine and were documented in the medical records.

The ASA score was the only performance score available preoperatively. It is routinely assessed by a trained anesthetist who reviews all available patient data and conducts a personal consultation with the patient the day before surgery. This score is mandatory for clearance for the upcoming surgery in our hospital and is thus routine.

For our analysis, the ASA score was taken from the abovementioned preoperative clearance in our patient records for the time before the initial surgery.

Postoperatively and before the commencement of adjuvant radiotherapy, the ECOG-PS was assessed by the treating radiation oncologist at initial patient presentation. The physician evaluated overall wellbeing and the ability of the patient to perform activities of daily life using the official ECOG-PS scale provided by the Eastern Cooperative Oncology Group [21].

The ACE-27 score was assessed retrospectively using all available medical records and the electronic documentation system. We extracted data concerning pre-existing conditions of various organ systems. Each comorbidity was scored from grade 0 (no comorbidity) to grade 3 (severe decompensation). The individual results were then added to give the final risk score for each patient. The maximum achievable score in the ACE-27 assessment is 4, where the presence of two comorbidities, each graded at level 2, is adequate for categorizing a patient into the highest risk group [13, 18, 19]. To assess the ACE-27 score we used the online freely available survey [22]. Although the primary tumor of the patient would be a part of the ACE-27 score, in this evaluation, it was deliberately excluded to maintain an assessment focused on comorbidities.

### Follow-up

Follow-up was calculated from the first day of the (chemo)radiation treatment. Time of death and cause of death were reported, as well as relapse either locally, locoregionally, nodally, or as distant metastases. All recurrences were histologically proven by needle biopsy or surgery.

### Statistics

The events of the survival endpoints were defined by the following: overall survival (OS) was indicated by death, local control (LC) was marked by primary tumor recurrence, and locoregional recurrence was characterized by any recurrence within the neck region.

Correlation analysis between different parameters was performed using Spearman’s rank correlation coefficient. Since there is no normal distribution for the measured parameters, additional information including median values and 95% confidence intervals were provided to reliably describe the data distributions. For survival analyses, Kaplan–Meier as well as uni- and multivariate Cox proportional hazard models were used. The Cox proportional hazard assumption was verified for each model using Schoenfeld residuals and log-rank test. Associations between categorical variables were assessed using the chi-squared test. The concordance index (C-Index) was used to assess predictive accuracy, indicating the likelihood that the patient with the higher predicted risk experiences the event (e.g., death or local recurrence) first.

For all statistical analyses a significance level of α = 0.05 was defined. The statistical analyses and data processing were performed using SPSS V26.0.0.1 (IBM Corp., Armonk, NY, USA) and R 4.0.4, employing libraries such as havel, survival, survminer, and ggplot2 (R Foundation for Statistical Computing, Vienna, Austria).

## Results

### Patient and treatment characteristics

All relevant demographic, clinical, and treatment-related data for the 302 consecutive patients treated in our department between 2012 and 2018 and included in the study are summarized in Table 1.

**Table 1:** Overview of patient and treatment characteristics

Description	Number/percentage
*Total number of patients*	302
*Gender*	
Female	75 (24.8%)
Male	227 (75.2%)
*Age at diagnosis*	Mean: 61 years (range 20–87)
*Tumor location*	
Oral cavity	61 (20.2%)
Oropharynx	151 (50%)
Hypopharynx	39 (12.9%)
Laryngeal	51 (16.9%)
*HPV status*	
HPV positive	70 (23.2%)
HPV negative	147 (48.7%)
Unknown	85 (28.1%)
*UICC stage (7th edition)*	
I	7 (2.3%)
II	30 (9.9%)
III	78 (25.8%)
IVa	181 (59.9%)
IVb	6 (2%)
*Surgical resection*	302 (100%)
With neck dissection	278 (92.1%)
Without neck dissection	24 (7.9%)
*Resection margins*	
Complete (R0)	116 (38.4%)
Close (< 5 mm)	107 (35.4%)
Positive (R1/2)	70 (23.2%)
Not defined	9 (3.0%)
*Extracapsular extension in lymph node metastasis*	26.5%
*Adjuvant radiotherapy*	302 (100%)
Median overall dose	64 Gy
Dose per day	2.0–2.2 Gy
Discontinued irradiation	9 (3%)

All patients were treated following the recommendations of pretherapeutic tumor boards. Concomitant chemotherapy was administered in 177 patients, mostly cisplatin/5-FU (45.0%). A full list of patient and treatment details is shown in Table 2 of the supplementary materials. A detailed distribution of individual performance scores, including stratification by HPV status, is provided in Tables 3 and 3.1 of the supplementary materials.

Overall survival, local control, and locoregional control analysis was performed for clinical parameters (age at diagnosis, gender, tumor stage, nodal stage, number of involved lymph nodes, ECE, HPV status, resection status, tumor grading) as well as for ECOG PS, ASA score, and ACE-27 score. Table 4 of the supplementary materials provides a summary of patient follow-up details, while Table 5 presents an overview of these parameters along with their corresponding hazard ratios and *p*-values derived from univariate analysis. Of 302 patients, ECOG-PS was documented before (chemo)radiotherapy for 214 patients. Preoperative ASA score was assessed in 229 patients. ACE-27 score was retrospectively assessed for all patients.

In our analysis, the univariate Cox analysis for clinical parameters demonstrated significant results for overall survival with factors including individual tumor stage (HR 1.3 [1.1–1.5]; *p* = 0.0015), number of removed pathological lymph nodes (HR 1.1 [1–1.1]; *p* < 0.001), extracapsular extension (HR 1.7 [1.2–2.4]; *p* = 0.002), HPV positivity (HR 0.44 [0.26–0.73]; *p* = 0.0015), preradiotherapy hemoglobin value (HR 0.9 [0.82–0.98]; *p* = 0.017), and preradiotherapy body mass index (HR 0.91 [0.85–0.97]; *p* = 0.005). For local control, HPV positivity was a significant factor (HR 0.22 [0.065–0.78]; *p* = 0.018), and for locoregional control, age at diagnosis (HR 0.94 [0.89–0.99]; *p* = 0.023) and tumor stage (HR 2.1 [1–4.2]; *p* = 0.038) were significant.

The performance scores exhibited significant results in terms of various clinical outcomes. ECOG-PS demonstrated a hazard ratio (HR) of 1.7 (95% CI: 1.3–2.4; *p* < 0.001) for overall survival, while the ASA score showed an HR of 2.6 (95% CI: 1.7–3.8; *p* < 0.001) for overall survival and an HR of 2.2 (95% CI: 1.0–4.6; *p* = 0.047) for local control. The ACE-27 score had an HR of 1.5 (95% CI: 1.3–1.7; *p* < 0.001) for overall survival and an HR of 0.4 (95% CI: 0.17–0.97; *p* = 0.043) for locoregional control.

Secondly, we assessed the statistical association between the performance scores ECOG-PS, ASA, and ACE-27, which are available as categorical variables, using the chi-square test. All three combinations were statistically significantly associated. ACE-27 and ASA demonstrated a *p*-value of < 0.001 (2.42 × 10^−5^) and ASA and ECOG-PS had a *p*-value of 0.021. The strongest association was observed between ACE-27 and ECOG-PS, which yielded a *p*-value of < 0.001 (1.17 × 10^−9^). Further, we tested the three scores for their prognostic impact with regard to overall survival in univariate Cox proportional hazard model analysis, which for ASA score yielded a log-rank test *p*-value of < 0.001 (1 × 10^−5^) and a concordance index (C-Index) [23] of 0.61, for ECOG-PS a *p*-value of 0.00147 and C-Index of 0.58, and for ACE-27 a *p*-value of < 0.001 (1 × 10^−5^) and a C-Index of 0.62, demonstrating a slightly better prognostic performance of ASA and ACE-27 compared to ECOG-PS.

Subsequent multivariate Cox proportional hazard analyses employing only the performance scores in relation to overall survival revealed varied prognostic performances reflected by C-Index and log-rank test *p*-values. The multivariate model including all three scores resulted in a C-Index of 0.66 and a *p*-value of < 0.001 (2 × 10^−4^). The model incorporating only ECOG and ASA scores yielded a C-Index of 0.62 and *p*-value of < 0.001 (5 × 10^−4^), while the model including ASA and ACE-27 scores achieved a C-Index of 0.66 and a *p*-value of < 0.001 (6 × 10^−7^). Hence, the simpler model containing ASA and ACE-27 only, not considering ECOG-PS, showed the best performance. This, along with the finding that ECOG-PS showed a very strong association with ACE-27, suggesting high informational redundancy between the two variables, led us to exclude ECOG-PS from further analyses.

**Fig. 1:**
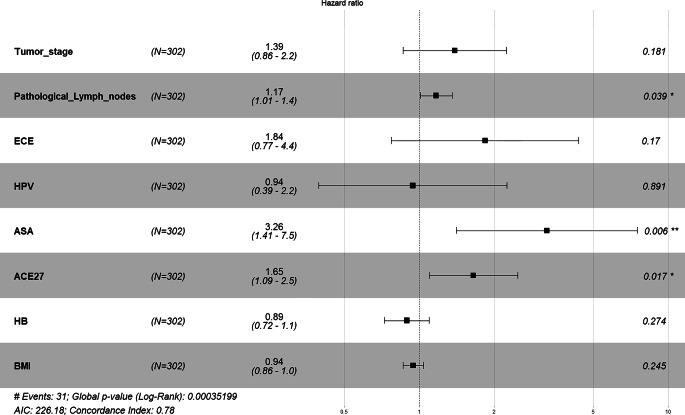
Forest plot of the multivariate Cox model of the parameters significant in univariate Cox analyses of overall survival. Number of pathological lymph nodes and the performance scores ASA and ACE-27 remained prognostically significant. ECOG-PS has been excluded due to a correlation with ACE-27 using Spearman rank correlation; furthermore, it showed a non-significant behavior in a direct comparison with ASA and ACE-27 in overall survival analysis. *ECE* Extracapsular extension, *HPV* human papillomavirus, *HB* hemoglobin prior to therapy, *BMI* Body-Mass-Index, * significant result (*p*<0.05), ** highly significant result (*p*<0.01)

**Fig. 2:**
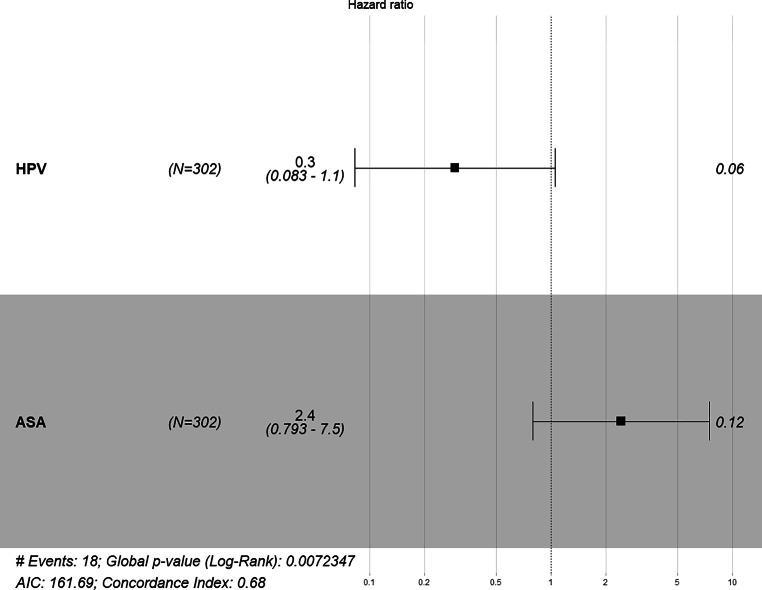
Forest plot using the multivariate Cox model for analyzing the hazard ratio for local recurrence

In the following multivariate Cox regression analyses (Fig. 1), only parameters significant in the univariate model were included. For overall survival, the number of pathological lymph nodes and the performance scores ASA and ACE-27 remained prognostically significant, with ASA score (HR 3.26 [1.41–7.5]; *p* = 0.006) and ACE-27 score (HR 1.65 [1.09–2.5]) being particularly notable. In the analysis of local recurrence, only HPV positivity remained significant. We applied Kaplan–Meier analysis between HPV status 0 or 1, which also led to a significant log-rank test (*p* = 0.01) showing a significant survival difference (Fig. 2). Notably, neither ASA score (HR 2.4 [0.793–7.5]; *p* = 0.12) nor ACE-27 score (HR 0.49 [0.21–1.2]; *p* = 0.1) remained significant for local or locoregional control, respectively (Fig. 6). For locoregional recurrence, only tumor stage remained highly significant (HR not provided).

The applied Kaplan–Meier analysis between tumor stages 1, 2, 3, and 4 did not lead to a significant log-rank test (*p* = 0.07). A significant locoregional recurrence difference between any tumor stages could therefore not be verified in our cohort.

**Fig. 3:**
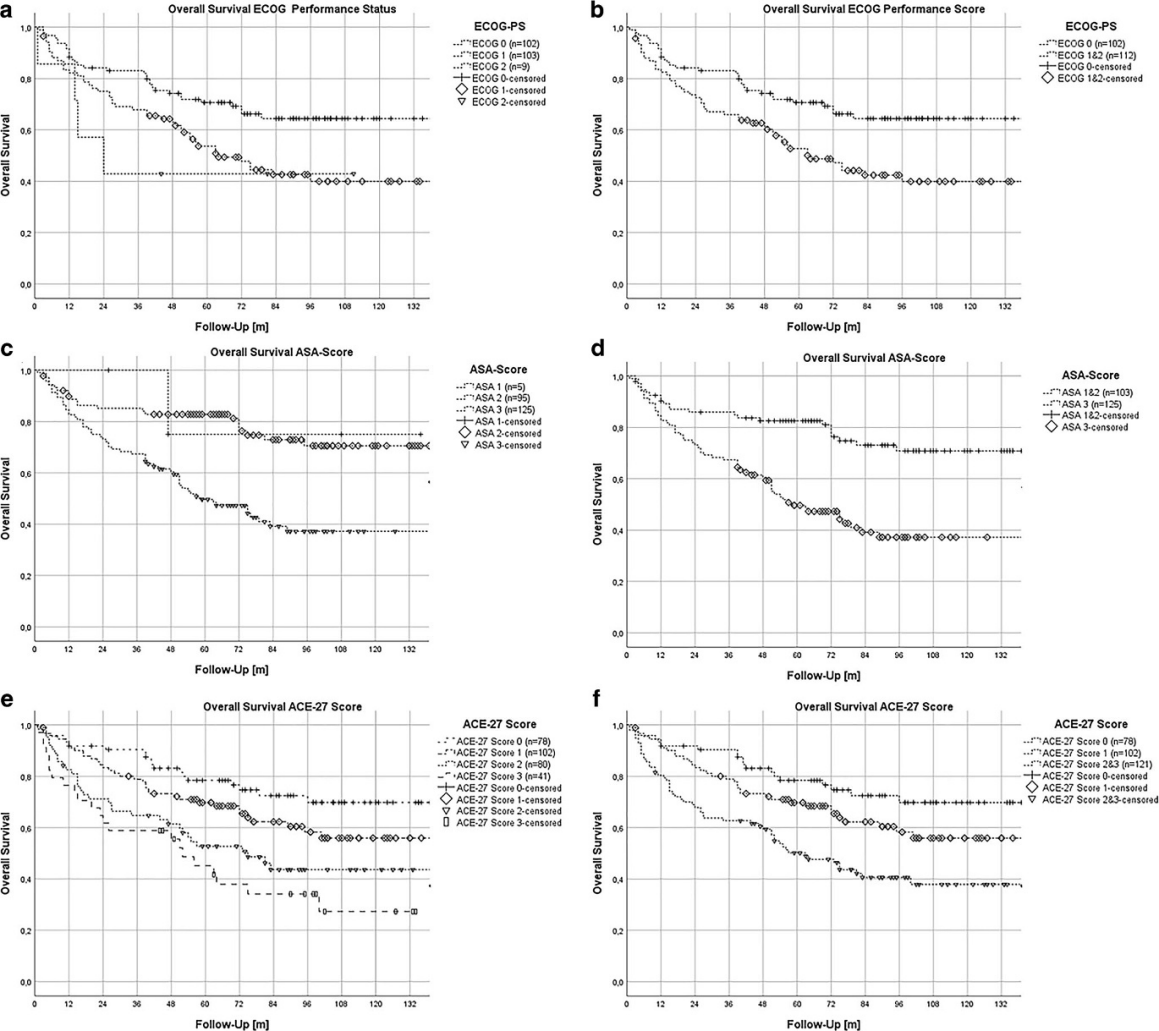
Kaplan–Meier plots (**a**–**f**) for OS separated by performance scores and stratified by performance score values with 95% confidence intervals (95%CI). **a,b **Kaplan–Meier plots for ECOG-PS and overall survival; pairwise comparison using log-rank test: **a **0 vs. 1 (*p* = 0.004); 0 vs. 2 (*p* = 0.088); 1 vs. 2 (*p* = 0.418); **b **pairwise comparison using log-rank test 0 vs. union of 1 and 2 (*p* = 0.003). Mean OS for ECOG-PS 0: 106.6 months (95% CI: 94.9–118.3 months); ECOG-PS 1: 78.1 months (95% CI: 65.8–90.4 months); ECOG-PS 2: 55.3 months (95% CI: 19.3–91.3 months); ECOG-PS 1 and 2: 77.1 months (65.2–89.1 months). **c,d **Kaplan–Meier plots for ASA score and overall survival; pairwise comparison using log-rank test: **c **1 vs. 2 (*p* = 0.843); 1 vs. 3 (*p* = 0.211); 2 vs. 3 (*p* < 0.001); **d **ASA score union of 1 and 2 vs. 3 (*p* ≤ 0.001). Mean OS for ASA score 1: 113.8 months (95% CI: 75.9–151.5 months); ASA score 2: 114.5 months (95% CI: 103.1–125.9 months); ASA score 3: 75.3 (95% CI: 64.2–68.3 months); ASA score 1 and 2: 115.0 months (103.9–126.0 months). **e,f **Kaplan–Meier plots for ACE-27 score on overall survival; pairwise comparison using log-rank test: ACE-27 score 0 vs. 1 vs. 2 vs. 3 resulting in **e **0 vs. 1 (*p* = 0.084), 0 vs. 2 (*p* = 0.001), 0 vs. 3 (*p* < 0.001), 1 vs. 2 (*p* = 0.063), 1 vs. 3 (*p* = 0.003), 2 vs. 3 (*p* = 0.236); **f **0 vs. 1 (*p* = 0.084); 1 vs. union of 2 and 3 (*p* = 0.008); 0 vs. union of 2 and 3 (*p* < 0.001). Mean OS for ACE-27 score 0: 115.9 months (95% CI: 103.8–128.0 months); ACE-27 score 1: 97.3 months (95% CI: 85.9–108.6 months); ACE-27 score 2: 79.5 months (95% CI: 64.8–94.2 months); ACE-27 score 3: 62.6 months (95% CI: 44.7–80.5 months); ACE-27 score 2 and 3: 74.3 months (95% CI: 62.6–86.0 months)

Figure 3 shows Kaplan–Meier plots for different performance scores (ASA, ACE-27, ECOG-PS). The log-rank tests were highly significant (*p* ≤ 0.001): in pairwise comparison, a highly significant difference could be verified between ASA scores 2 and 3 (*p* < 0.001) and between ACE-27 scores 0 and 3 (*p* < 0.001) and 0 and 2 (*p* = 0.001). Pairwise comparison between ACE 27 scores 0 and 1 was not significant in our analysis (*p* = 0.084) but did show a trend.

Due to the small number of patients in various comorbidity score groups, we combined ACE-27 scores of 2 and 3 as well as ASA scores 1 and 2 and ECOG scores 1 and 2 for further analysis. This grouping showed almost significant differences for ACE-27 high-risk scores compared to scores 0 and 1. For ASA score, combining scores 1 and 2 resulted in a highly significant difference to score 3 (*p* < 0.001). Similarly, combined ECOG scores 1 and 2 showed a significant difference when compared with ECOG score 0 (*p* = 0.003).

**Fig. 4:**
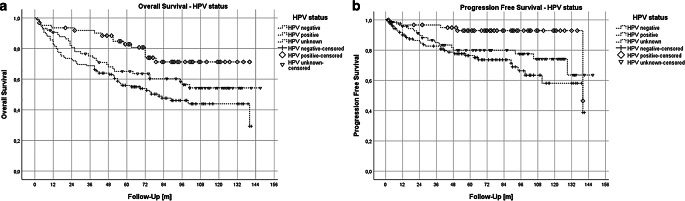
Kaplan–Meier plots for OS and PFS stratified by HPV status. **a** Kaplan–Meier plot for OS with regard to HPV status; pairwise comparison using log-rank test: HPV neg. vs. HPV pos. (*p* ≤ 0.001); HPV neg. vs. HPV unknown (*p* = 0.126); HPV pos. vs. HPV unknown (*p* = 0.046). **b** Kaplan–Meier plot for PFS with regard to HPV status; pairwise comparison using log-rank test: HPV neg. vs. HPV pos. (*p* = 0.001); HPV neg. vs. HPV unknown (*p* = 0.179); HPV pos. vs. HPV unknown (*p* = 0.035)

Figure 4 displays Kaplan–Meier plots for overall survival (OS) and progression-free survival (PFS) stratified by HPV status. Log-rank tests revealed significant differences in OS and PFS between HPV-negative and HPV-positive HNSCC patients (*p* < 0.001 for OS and *p* = 0.001 for PFS), and between HPV-positive patients and those with unknown HPV status (OS *p* = 0.046 and PFS *p* = 0.035). Kaplan–Meier analysis indicated a mean OS of 80.3 months for HPV-negative and 112.2 months for HPV-positive tumors.

**Fig. 5:**
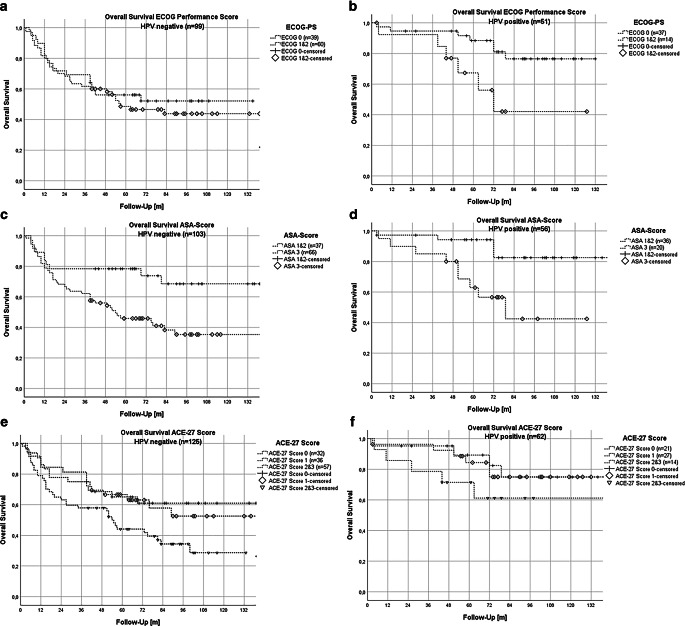
Kaplan–Meier plot for OS separated by HPV status and stratified by comorbidity scores. Kaplan–Meier plots (**a**–**f**) for OS separated by HPV status and stratified by different performance scores with 95% confidence intervals (95%CI), i.e., ECOG-PS, ASA score, and ACE-27 score. **a,b** Kaplan–Meier plots for OS separated by HPV status and stratified by ECOG-PS; pairwise comparison using log-rank test: **a **HPV negative: ECOG-PS 0 vs. 1 and 2 (*p* = 0.548); **b **HPV positive: ECOG-PS 0 vs. 1 and 2 (*p* = 0.017); mean OS for ECOG 0: HPV neg. 81.9 months (95% CI: 67.8–100.1 months), HPV pos. 113.6 months (95% CI: 101.2–126.0 months). Mean OS for ECOG 1 and 2: HPV neg. 77.1 months (95% CI: 61.8–92.4 months), HPV pos. 82.2 months (95% CI: 56.9–107.4 months). **c,d **Kaplan–Meier plot for OS separated by HPV status and stratified by ASA score; pairwise comparison using log-rank test: **c **HPV negative: ASA score 1 and 2 vs. 3 (*p* = 0.01); **d **HPV positive: ASA score 1and 2 vs. 3 (*p* = 0.005). Mean OS for ASA score 1 and 2: HPV neg. 105.3 months (95% CI: 86.1–124.5 months); HPV pos. 125.2 months (95% CI: 113.2–137.3 months). Mean OS for ASA score 3: HPV neg. 71.2 months (95% CI: 57.1–85.3 months), HPV pos. 82.4 months (95% CI: 61.1–103.7 months). **e,f **Kaplan–Meier plots for OS separated by HPV status and stratified by ACE-27 score; pairwise comparison using log-rank test: **e **HPV negative: ACE-27 score 0 vs. 1 (*p* = 0.646); 0 vs. 2 and 3 (*p* = 0.029); 1 vs. 2 and 3 (*p* = 0.057); **f **HPV positive: ACE-27 score 0 vs. 1 (*p* = 0.868); 0 vs. 2 and 3 (*p* = 0.203); 1 vs. 2 and 3 (*p* = 0.233). Mean OS for ACE-27 score 0: HPV neg. 97.9 months (95% CI: 78.3–117.4 months); HPV pos. 115.9 months (95% CI: 98.3–133.6 months). Mean OS for ACE-27 score 1: HPV neg. 91.2 months (95% CI: 71.6–110.8 months), HPV pos. 117.7 months (95% CI: 101.8–133.6 months). Mean OS for ACE-27 score 2and 3: HPV neg. 64.4 months (95% CI: 50.3–78.2 months); HPV pos. 98.2 months (95% CI: 68.7–127.7 months)

Figure 5 further separates these findings by comparing performance scores stratified by HPV status. For ECOG-PS, only HPV-positive tumors showed a significant difference in OS between ECOG-PS 0 and ECOG-PS 1 and 2 (*p* = 0.017), while results for HPV-negative tumors were not significant (*p* = 0.548). For ASA score, significant differences in OS were observed for both HPV-negative (*p* = 0.01) and HPV-positive tumors (*p* = 0.005) between ASA 1 and 2 and ASA 3. For ACE-27 score, significant results were noted for HPV-negative tumors between score 0 and high-risk scores 2 and 3 (*p* = 0.029), and a nearly significant difference was noted between score 1 and high-risk scores 2 and 3 (*p* = 0.057). HPV-positive tumors showed only a positive trend in these comparisons.

**Fig. 6:**
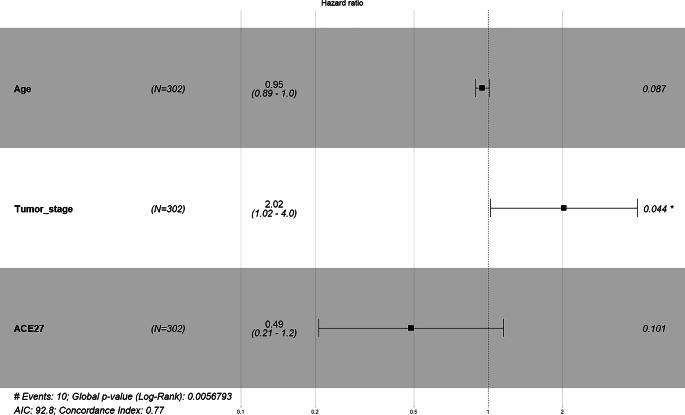
Forest plot using a multivariate Cox model analyzing hazard ratio for locoregional recurrence. Tumor stage status remained prognostically significant (*p* = 0.044)

### Follow-up

Median follow-up was 61.8 months (range 1–150), and 97 out of 302 patients died. Of these deaths, 42 deaths were tumor related, and other causes were comorbidities (*n* = 40), therapy related (*n* = 2), second primary tumors (*n* = 8), and unknown (*n* = 5). 35 patients (11.6%) had local failure, locoregional failure was seen in 70 patients (23.2%), isolated lymph node failure in 36 patients (11.9%). 39 patients (12.9%) developed distant metastases. The 3‑/5-year OS and DFS rates of the overall cohort were 70.5% and 60.2% and 64.7% and 57.6% respectively.

In the subgroup of HPV-positive HNSCC patients (*n* = 70), 3 experienced local failure, 2 with locoregional recurrence, and 2 patients had distant failure, resulting in a 3-/5-year OS of 88.6% and 78.1%, respectively. Table 4 provides further details.

## Discussion

The purpose of this study was to evaluate whether additional preoperative or objective indices other than ECOG-PS can help in guiding the treatment decision by stratifying patients according to their individual risk group.

### ECOG performance status

The prognostic significance of ECOG-PS in predicting overall survival (OS) across diverse cancer types has been substantiated in manifold studies. Our analysis indicated that while the ECOG-PS significantly affected OS in univariate analysis, it did not retain its significance in multivariate models. Wang et al. reported similar results, noting that ECOG-PS, alongside the Charlson comorbidity index (CCI), provided prognostic insights for OS but not for cancer-specific survival (CSS) in a cohort of 600 HNSCC patients [2]. These results confirm the association with overall survival (OS) but did not apply to more cancer-specific endpoints such as cancer-specific survival (CSS) in their analysis or progression-free survival (PFS) in ours. However, ECOG-PS has some limitations, including high interrater variability due to the lack of clear cut-offs between different categories, as shown by Datta et al. [24]. Here, preoperative or additional ECOG values for the same patient would be interesting to examine this interrater variability, which, however, was not possible in our analysis. Additionally, the ECOG performance status is usually assessed preradio(chemo)therapy and might be influenced by prior surgery that could impair its predictive value. Unfortunately, we do not have preoperative ECOG-PS data to verify this in our study. However, with 102 (33.8%) patients rated ECOG 0 and 103 (34.1%) rated ECOG 1, the scores might appear better than actual patient conditions suggest. For instance, among those rated ECOG 0, 40 had an ACE-27 score of 1, 17 had a score of 2, and 2 had a score of 3, indicating varying levels of comorbidity. A similar pattern is observed with the ASA score, where 4, 45, and 45 patients had scores of 1, 2, and 3, respectively.

### ASA score

Within our cohort, the ASA score was notably predictive in univariate analyses for overall survival (OS) and local recurrence, and retained its prognostic relevance for OS in multivariate models, outperforming the predictive power of ECOG-PS. These results align with prior research. Hackett et al. have confirmed the prognostic utility of the ASA score, demonstrating its strong and independent correlation with postoperative complications and mortality across various procedures [16]. In the context of upper tract urothelial cancer, Yuan et al. and Kang et al. demonstrated that elevated ASA scores correlated with diminished overall and cancer-specific survival [25, 26], thus proving the relevance of this score. Interestingly, while most previous studies have assessed the ASA score postoperatively following a single surgical intervention without subsequent radiochemotherapy, our analysis demonstrates that the ASA score retains its predictive value even after surgery followed by postoperative radiochemotherapy. Despite these findings, the application of the ASA score in head and neck squamous cell carcinoma (HNSCC) remains contentious. Diverse studies have conflicting results regarding its prognostic accuracy, highlighting the need for further investigation into its comparative effectiveness and potential limitations, particularly its general preoperative application without adjustments for patient age and specific cancer types [27].

### ACE-27 score

In our study, the ACE-27 score, the most objective indicator of performance status utilized, was significantly associated with overall survival (OS) in multivariate analyses, supporting existing evidence of its predictive capability in patients with head and neck squamous cell carcinoma (HNSCC) [28].

Milne et al. [29] further validated the ACE-27’s superior predictive accuracy for 2‑year mortality compared to the TNM staging system, along with its correlation with hospitalization duration and complication rates. In our analysis, the ACE-27 score outperformed tumor stage in predicting OS, underscoring its considerable prognostic value.

Aligning with findings from Datema et al. [30], an ACE-27 score of 3 was comparable to the short-term mortality risk associated with T4 or N2 tumors. Our findings showed a mean OS of 64.2 months for T4 tumors and 61.0 months for patients with an ACE-27 score of 3. Moreover, T1 and T2 tumors with an ACE-27 score of 3 resulted in mean OS durations of 79.2 and 52.3 months, respectively. Binder et al. [11], supported these results, indicating that an ACE-27 score of 2 or higher is associated with reduced overall survival (OS) and a heightened recurrence risk, thereby echoing our results. This underscores the significant impact of comorbidities on OS. However, unlike the findings of Wen et al. [31], our study did not show a discernible influence of comorbidities on cancer-specific survival (CSS) or progression-free survival (PFS).

The ACE-27 score has demonstrated its effectiveness in various cancers, with research indicating that higher scores are associated with decreased overall survival in endometrial cancer, as noted by Tian et al. [32], and affecting median overall survival in myelodysplastic syndromes, as reported by Daver et al. [33].

Furthermore, even during radiochemotherapy, Monteiro et al. [34] underscored the ACE-27 score’s utility in identifying patients at a higher risk of significant toxicities, thus reinforcing its value in clinical settings. Since toxicity data were not available for our analysis, we were unable to examine this aspect. However, having used the ACE-27 score to assess overall survival, the logical next step would be to investigate whether patients with higher comorbidity burdens experience increased toxicities during postoperative radiochemotherapy. This would help us to move towards a more individualized evidence-based approach to therapy that is tailored to patients’ specific comorbidity profiles.

In this context, the deliberate exclusion of the primary tumor diagnosis as an item from the ACE-27 score itself is particularly relevant. It allows for an undiluted analysis of comorbidity impacts on patient outcomes, independently of direct tumor effects. This focused approach enhances our understanding of the sole influence of comorbidities on crucial outcomes like overall survival and locoregional control. These data collectively affirm the ACE-27 index as a vital tool for comorbidity evaluation in oncological practice and underscore its utility in prognostic stratification, making it the recommended comorbidity tool for cancer patients in the UK.

Although the performance scores mentioned above provide a comprehensive assessment of comorbidities, there is a need for further research to determine whether certain comorbidities among these scores, such as cardiovascular disease, diabetes mellitus, or neurological conditions, have a greater impact on survival in conjunction with TNM staging, resulting in even more precise survival predictions.

In a subanalysis of the patients with known HPV status, our results indicate that HPV-positive HNSCC patients generally experience better overall survival (OS) and progression-free survival (PFS), confirming the reliability of our data in reflecting trends in HNSCC [35–37]. Additionally, patients with HPV-positive tumors tend to have better comorbidity scores than those with HPV-negative ones, who often show moderate to severe scores, suggesting worse overall health and more severe comorbidities. These findings suggest that the observed differences between HPV-positive and HPV-negative HNSCC patients may be attributed to a combination of distinct biological characteristics as well as health behaviors. Notably, there is no significant age difference between the HPV-positive and HPV-negative HNSCC patients, with mean age of 61 years (interquartile range [IQR] 54–67 years) and 60 years (IQR 54–68 years), respectively. When analyzing overall survival (OS) stratified by risk scores, HPV status yields varied results. The ECOG score is not a predictive parameter for OS in HPV-negative patients, whereas it does influence OS in HPV-positive tumors. Wagner et al. reported comparable findings, observing that HPV-positive tumors with an ECOG-PS of less than 1 demonstrated the best OS outcomes for squamous cell carcinoma of unknown primary in the head and neck region [38]. In contrast, for the ACE-27 score, only HPV-negative tumors show a significant difference between scores of 0 and 2 and 3 combined. Meanwhile, the mean OS values for ACE-27 scores of 0 and 1 are similar for both HPV-negative and HPV-positive tumors, suggesting that mild comorbidities (ACE-27 score 1) may not significantly impact OS compared to no comorbidities (ACE-27 score 0) but they do when compared to moderate and severe comorbidities indicated by ACE-27 scores of 2 and 3 combined. Our analysis of HPV status is constrained by the number of patients with confirmed HPV status, since our study includes all types of HNSCC, not just oropharyngeal cancers.

There are several limitations to our study. First, the retrospective nature of our design may pose challenges in accurately assessing each comorbidity based on patient records, with the possibility of overlooking unrecorded comorbidities. In the worst-case scenario, this could lead to a misclassification of patients. However, to mitigate these limitations, we collected data from multiple documents from various departments in our clinic, and all comorbidities were preoperatively recorded by a skilled anesthetist and re-recorded before radio(chemo)therapy. Although ECOG-PS and ASA score were not assessable for every patient, potentially influencing the results, we took significant steps to reduce this potential error by utilizing a large cohort size. Additionally, we did not have access to preoperative ECOG-PS values, which would have allowed for a more comprehensive comparison between pre- and postoperative ECOG status.

This highlights the vital importance of consistently recording and documenting scores that reflect the general condition at each treatment decision. However, the availability of preoperative ASA and ACE-27 scores, which are more objective, enabled us to perform a comparative analysis. Therefore, we consider the absence of preoperative ECOG-PS data a minor limitation in the context of our study.

Further correlation between comorbidity and posttherapeutic toxicity was not performed, as only pretherapeutic data were available. Another limitation is that we did not record the CCI, another well-established comorbidity risk score in HNSCC [39], for further comparison. However, since this index covers fewer comorbidities and does not quantify comorbidity severity, we do not consider this a major limitation of our study.

Despite certain limitations, our study has noteworthy strengths. With a sample size of 302 patients and a median follow-up of 62 months, our study provides significant statistical power. Moreover, our cohort represents a typical clinical population rather than a highly selective and rigorously screened group. Notably, our multivariate analysis and Kaplan–Meier analysis demonstrate a significant association between HPV positivity and overall survival, aligning with current research and establishing the reliability and robustness of our cohort. The mean overall survival figures for the three assessed comorbidity levels, further stratified by HPV status, provide valuable insights into how biological factors and health behaviors affect patient outcomes.

## Conclusion

Our results resonate with extant literature that attributes a more pronounced influence of comorbidities on overall survival than on cancer-specific metrics, predominantly due to an increased likelihood of non-cancer-related mortality. The ACE-27 and ASA scores, with their more objective assessments, seem to offer greater accuracy than the ECOG performance status for predicting overall survival. This supports their inclusion as additional metrics in clinical decision-making processes for cancer treatment.

## Supplementary Information

Table 2: Patients and treatment characteristics, Table 3: Insights on Performance Scores and HPV-status, Table 3.1: Analysis and results of performance scores and mean Overall Survival stratified by HPV status, Table 4: Patient Follow-up details, Table 5: Analysis and results of univariate analysis for Overall Survival, Local Recurrence and Locoregional Recurrence

## Data Availability

All data generated or analyzed during this study are included in this published article and its supplementary information files

## Abbreviations

ACE-27 score: Adult Comorbidity Evaluation-27 score
ASA score: American Society of Anesthesiologists score
CDDP: Cisplatin, cis-diamminedichloroplatinum(II)
CSS: Cancer-specific survival
DFS: Disease-free survival
DSS: Disease-specific survival
ECE: Extracapsular extension
ECOG-PS: Eastern Cooperative Oncology Group performance status
5‑FU: 5‑Fluorouracil
HNSCC: Head and neck squamous cell carcinoma
HPV: Human papillomavirus
IMRT: Intensity-modulated radiotherapy
IPSS: International Prognostic Scoring System
KPS: Karnofsky performance status
LMU: Ludwig Maximilian University Munich
MMC: Mitomycin C
OS: Overall survival
PFS: Progression-free survival
UICC: Union for International Cancer Control
