# Supplementary material for: Adjuvant (chemo)radiotherapy for patients with head and neck cancer: can comorbidity risk scores predict outcome?
Source: Strahlenther Onkol. 2024 Sep 2;200(12):1025–37. doi: 10.1007/s00066-024-02282-y (PMC11588950; doi:10.1007/s00066-024-02282-y)
Supplement: Supplementary file 1 — Table 2: Patients and treatment characteristics, Table 3: Insights on Performance Scores and HPV-status, Table 3.1: Analysis and results of performance scores and mean Overall Survival stratified by HPV status, Table 4: Patient Follow-up details, Table 5: Analysis and results of univariate analysis for Overall Survival, Local Recurrence and Locoregional Recurrence [file 66_2024_2282_MOESM1_ESM.docx]

**Table 2 - Patients and treatment characteristics**

| **Table 2. Patients and treatment characteristics** | | | | | | | |
| --- | --- | --- | --- | --- | --- | --- | --- |
|  |  | **Patients (=n)** | **Percentage (in %)** |  |  | **Patients (=n)** | **Percentage (in %)** |
| **Sex** | Male | 227 | 75.2 | **Metastasis (M)** | No | 302 | 100.0 |
|  | Female | 75 | 24.8 |  | Yes | 0 | 0 |
| **Age** | <45 | 11 | 3.6 | **Resection margin (R)** | Negative | 116 | 38.4 |
|  | 45-54 | 66 | 21.9 |  | Close margin (<5mm) | 107 | 35.4 |
|  | 55-64 | 114 | 37.7 |  | Positive | 70 | 23.2 |
|  | 65-74 | 88 | 29.1 |  | Unkonwn | 9 | 2.9 |
|  | >75 | 23 | 7.6 | **Extracapsular Extension (ECE)** | No ECE | 207 | 68.5 |
| **Smoking history** | Yes | 288 | 95.4 |  | Yes | 80 | 26.5 |
|  | no | 14 | 4.6 |  | Unknown | 15 | 4.9 |
|  | unknown | 0 | 0 | **Irradiation dose** | Median | 64 Gy |  |
| **Anemia** | normal Hb | 157 | 52.0 |  | <60 Gy | 9 | 3.0 |
|  | Anemia | 141 | 46.7 |  | 60-63,9 Gy | 8 | 2.6 |
|  | unknown | 4 | 1.3 |  | 64 - 66Gy | 285 | 94.4 |
| **HPV/p16** | Positive | 70 | 23.2 |  | >66Gy | 0 | 0 |
|  | Negative | 147 | 48.6 | **Irradiation completed as planned** | Yes | 293 | 97.0 |
|  | unknown | 85 | 28.1 |  | No | 9 | 3.0 |
| **Localisation** | Oral cavity | 61 | 20.2 | **Irradiation technique** | 3D | 245 | 81.1 |
|  | Oropharynx | 151 | 50.0 |  | IMRT | 57 | 18.9 |
|  | Hypopharynx | 39 | 12.9 | **Irradiation PTV Boost Volume** | <1000ccm | 40 | 13.2 |
|  | Larynx | 51 | 16.9 |  | 101-200 ccm | 95 | 31.5 |
| **UICC** | I | 7 | 2.3 |  | 201-300 ccm | 61 | 20.2 |
|  | II | 30 | 9.9 |  | 301-400ccm | 83 | 27.5 |
|  | III | 78 | 25.8 |  | >400 ccm | 23 | 7.6 |
|  | IVa | 181 | 59.9 | **Chemotherapy concomitant** | No Chemotherapy | 125 | 41.3 |
|  | IVb | 6 | 2.0 |  | Cis/ 5-FU (ARO) | 136 | 45.0 |
| **Primary Tumor (T)** | 1 | 59 | 19.6 |  | Cisplatin weekly 40mg/m2 | 9 | 2.9 |
|  | 2 | 123 | 40.7 |  | Cetuximab | 2 | 0.6 |
|  | 3 | 71 | 23.5 |  | 5-FU / MMC | 10 | 3.0 |
|  | 4 | 49 | 16.2 |  | MMC alone | 20 | 6.6 |
| **lymph Nodes (N)** | 0 | 89 | 29.5 |  | | | |
|  | 1 | 58 | 19.2 |  |  |  |  |
|  | 2a | 23 | 7.6 |  |  |  |  |
|  | 2b | 85 | 28.1 |  |  |  |  |
|  | 2c | 42 | 13.9 |  |  |  |  |
|  | 3 | 5 | 1.6 |  |  |  |  |

**Table 3 - Performance scores**

| **Table 3. Performance scores and HPV status** | | | | | | |
| --- | --- | --- | --- | --- | --- | --- |
|  | | **All patients n=302** | | **HPV negativ  (n=147)** | **HPV positiv  (n=70)** | **HPV unknown  (n=85)** |
| **Score** | | **Patients (=n)** | **Mean OS in months (95%CI)** | **Patients (=n)** | **Patients (=n)** | **Patients (=n)** |
| **ECOG-PS** | 0 | 102 (33.8%) | 106.6 (94.9 - 118.3) | 43 (29.3%) | 39 (55.7%) | 20 (23.5%) |
|  | 1 | 103 (34.1%) | 78.1 (65.8 - 90.4) | 66 (44.9%) | 13 (18.6%) | 24 (28.2%) |
|  | 2 | 9 (3.0%) | 55.3 (19.3 - 91.3) | 6 ( 4.1%) | 2 ( 2.9%) | 1 ( 1.2%) |
|  | 1&2 | 112 (37.1) | 77.1 (65.2 - 89.1) | 72 (49.0%) | 15 (21.5%) | 25 (29.4%) |
|  | unknown | 88 (40.4%) | 100.6 (88.5 - 112.7) | 32 (21.8%) | 16 (22.9%) | 40 (47.1%) |
| **ASA-Score** | 1 | 5 (1.7%) | 113.8 (75.9 - 151.5) | 0 | 4 ( 5.7%) | 1 ( 1.2%) |
|  | 2 | 98 (32.5%) | 114.5 (103.1 - 125.9) | 41 (27.9%) | 33 (47.1%) | 24 (28.6%) |
|  | 3 | 126 (41.7%) | 75.3 (64.2 - 68.3) | 81 (55.1%) | 24 (34.3%) | 21 (25.0%) |
|  | 1&2 | 103 (34.2%) | 115.0 (103.9 - 126.0) | 41 (27.9%) | 37 (52.8%) | 25 (29.8%) |
|  | unknown | 74 (25%) | 93.2 (79.4 - 107.0) | 25 (17.0%) | 9 (12.9%) | 38 (45.2%) |
| **ACE-27 Score** | 0 | 78 (25.8%) | 115.9 (103.8 - 128.0) | 36 (24.5%) | 21 (30.0%) | 21 (24.7%) |
|  | 1 | 102 (33.8%) | 97.3 (85.9 - 108.6) | 42 (28.6%) | 30 (42.9%) | 30 (35.3%) |
|  | 2 | 81 (26.8%) | 79.5 (64.8 - 94.2) | 47 (32.0%) | 12 (17.1%) | 22 (25.9%) |
|  | 3 | 41 (13.6%) | 62.6 (44.7 - 80.5) | 22 (15.0%) | 7 (10.0%) | 12 (14.1%) |
|  | 2&3 | 122 (40.4%) | 71.7 (58.1 - 85.3) | 69 (47.0%) | 19 (27.1%) | 32 (30.0%) |

**Table 3.1 - Performance Scores with mean Overall Survival stratified by HPV status**

| **Table 3. Performance scores stratified by HPV status with mean Overall Survival** | | | | | | | |
| --- | --- | --- | --- | --- | --- | --- | --- |
|  | | **All patients (n=302)** | | **HPV negativ (n=147)** | | **HPV positiv (n=70)** | |
| **Score** | | **Patients (=n)** | **Mean OS in months (95%CI)** | **Patients (=n)** | **Mean OS in months (95%CI)** | **Patients (=n)** | **Mean OS in months (95%CI)** |
| **ECOG-PS** | 0 | 102 (33.8%) | 106.6 (94.9 - 118.3) | 43 (29.3%) | 81,9 (67,8 - 100,1) | 39 (55.7%) | 113.6 (101.2 - 126.0) |
|  | 1&2 | 112 (37.1) | 77.1 (65.2 - 89.1) | 72 (49.0%) | 77.1 (61.8 - 92.4) | 15 (21.5%) | 82.2 (56.9 - 107.4) |
| **ASA-Score** | 1&2 | 103 (34.2%) | 115.0 (103.9 - 126.0) | 41 (27.9%) | 105.3 (86.1 - 124.5) | 37 (52.8%) | 125.2 (113.2 - 137.3) |
|  | 3 | 126 (41.7%) | 75.3 (64.2 - 68.3) | 81 (55.1%) | 71.2 (57.1 - 85.3) | 24 (34.3%) | 82.4 (61.1 - 103.7) |
| **ACE-27 Score** | 0 | 78 (25.8%) | 115.9 (103.8 - 128.0) | 36 (24.5%) | 97.9 (78.3 - 117.4) | 21 (30.0%) | 115.9 (98.3 - 133.6) |
|  | 1 | 102 (33.8%) | 97.3 (85.9 - 108.6) | 42 (28.6%) | 91.2 (71.6 - 110.8) | 30 (42.9%) | 117.7 (101.8 - 133.6) |
|  | 2&3 | 122 (40.4%) | 71.7 (58.1 - 85.3) | 69 (47.0%) | 64.4 (50.3 - 78.2) | 19 (27.1%) | 98.2 (68.7 - 127.7) |

**Table 4 - Patient Follow-up**

| **Table 4. Patient Follow-Up** | | | |
| --- | --- | --- | --- |
|  |  | **Patients (=n)** | **Percentage** |
| **Causes of death** | Comorbidities | 40 | 13.2 |
|  | Tumor related | 42 | 13.9 |
|  | Therapy-associated | 2 | 0.6 |
|  | Second primary | 8 | 2.6 |
|  | Unknown | 5 | 1.7 |
| **Local Relapse** | No | 276 | 91.4 |
|  | Yes | 35 | 11.6 |
| **Locoregional Relapse** | No | 232 | 76.8 |
|  | Yes | 70 | 23.2 |
| **Nodal relapse** | No | 266 | 88.1 |
|  | Yes | 36 | 11.9 |
| **Distant metastasis** | No | 263 | 87.1 |
|  | Yes | 39 | 12.9 |

**Table 5 - Univariate analysis**

|  | **Overall survival** | | **Local Recurrence** | | **Locoreg. Recurrence** | |
| --- | --- | --- | --- | --- | --- | --- |
|  | **HR (95% CI for HR)** | **p-value** | **HR (95% CI for HR)** | **p-value** | **HR (95% CI for HR)** | **p-value** |
| **Age at diagnosis** | 1 (1-1) | 0.15 | 0.98 (0.95-1) | 0.21 | 0.94 (0.89-0.99) | 0.023* |
| **Gender** | 0.83 (0.57-1.2) | 0.35 | 0.98 (0.46-2.1) | 0.96 | 1.3 (0.33-4.9) | 0.73 |
| **Tumor stage** | 1.3 (1.1-1.5) | 0.0015* | 1.1 (0.8-1.6) | 0.48 | 2.1 (1-4.2) | 0.038* |
| **Nodal stage** | 1.1 (0.91-1.3) | 0.3 | 0.8 (0.55-1.2) | 0.26 | 0.6 (0.28-1.3) | 0.19 |
| **Number of path. ln** | 1.1 (1-1.1) | <0.001* | 0.98 (0.84-1.1) | 0.79 | 0.8 (0.52-1.2) | 0.32 |
| **ECE** | 1.7 (1.2-2.4) | 0.002* | 1.4 (0.68-3.1) | 0.34 | 0.93 (0.19-4.5) | 0.93 |
| **HPV** | 0.44 ( 0.26-0.73) | 0.0015* | 0.22 (0.065-0.78) | 0.018* | 0.43 (0.086-2.1) | 0.3 |
| **Resection status** | 0.96 (0.69-1.3) | 0.81 | 1.3 (0.62-2.7) | 0.5 | 6.9 (0.86-55) | 0.069 |
| **Tumor grading** | 1.1 (0.81-1.5) | 0.54 | 0.73 (0.41-1.3) | 0.29 | 0.62 (0.22-1.8) | 0.38 |
| **ECOG-PS** | 1.7 (1.3-2.4) | <0.001* | 0.81 (0.4-1.6) | 0.54 | 0.26 (0.056-1.2) | 0.091 |
| **ASA-Score** | 2.6 (1.7-3.8) | <0.001* | 2.2 (1-4.6) | 0.047* | 1.3 (0.39-4.3) | 0.68 |
| **ACE-27 Score** | 1.5 (1.3-1.7) | <0.001* | 0.99 (0.71-1.4) | 0.95 | 0.4 (0.17-0.97) | 0.043* |
| **Hb before RT** | 0.9 (0.82-0.98) | 0.017* | 0.95 (0.79-1.1) | 0.61 | 0.99 (0.69-1.4) | 0.96 |
| **BMI before RT** | 0.91 (0.85-0.97) | 0.005* | 0.99 (0.89-1.1) | 0.93 | 0.93 (0.74-1.2) | 0.55 |

Table 5: Univariate analysis of different parameters on overall survival, local recurrence and locoregional recurrence, * significant result
